# Multicomponent Spatial-Temporal Graph Attention Convolution Networks for Traffic Prediction with Spatially Sparse Data

**DOI:** 10.1155/2021/9134942

**Published:** 2021-12-23

**Authors:** Shaohua Liu, Shijun Dai, Jingkai Sun, Tianlu Mao, Junsuo Zhao, Heng Zhang

**Affiliations:** ^1^School of Electronic Engineering, Beijing University of Posts and Telecommunications, Beijing 100876, China; ^2^Beijing Key Laboratory of Mobile Computing and Pervasive Device, Institute of Computing Technology, Chinese Academy of Sciences, Beijing 100190, China; ^3^Institute of Software Chinese Academy of Sciences, Beijing 100190, China

## Abstract

Predicting traffic data on traffic networks is essential to transportation management. It is a challenging task due to the complicated spatial-temporal dependency. The latest studies mainly focus on capturing temporal and spatial dependencies with spatially dense traffic data. However, when traffic data become spatially sparse, existing methods cannot capture sufficient spatial correlation information and thus fail to learn the temporal periodicity sufficiently. To address these issues, we propose a novel deep learning framework, Multi-component Spatial-Temporal Graph Attention Convolutional Networks (MSTGACN), for traffic prediction, and we successfully apply it to predicting traffic flow and speed with spatially sparse data. MSTGACN mainly consists of three independent components to model three types of periodic information. Each component in MSTGACN combines dilated causal convolution, graph convolution layer, and the weight-shared graph attention layer. Experimental results on three real-world traffic datasets, METR-LA, PeMS-BAY, and PeMSD7-sparse, demonstrate the superior performance of our method in the case of spatially sparse data.

## 1. Introduction

Traffic prediction is one of the most essential tasks in the Intelligent Transportation System [1]. The goal of this task is to predict the future traffic conditions (e.g., traffic speed and traffic volume) by analyzing the historical traffic data. Accurate and timely traffic prediction is essential to many real-world applications. For example, if traffic data could be predicted accurately in advance, the transportation department can dynamically adjust the time of traffic lights; moreover, the navigation system can change the route in time to reduce congestion. However, traffic prediction is very challenging because of the dynamic spatial correlations and nonlinear temporal correlations. Early traffic prediction methods [2, 3] can be divided into classic statistical methods and machine learning models, which are limited by the stationarity assumption and fail to capture the spatial correlations.

Recently, many deep learning models have been proposed for traffic prediction. For spatial modeling, graph convolutional neural networks (GCN) [4–6] are widely used in graph-based data. Diffusion convolutions [7] and attention mechanism [8–13] are also adopted by researchers to capture spatial dependencies. From the perspective of periodicity, some methods use time information of sample data as additional input features [5, 7] to learn periodic information, and some attempts [8, 14, 15] divide the data and model into multiple components to capture the correlations under different periods. However, existing methods have the following shortcoming.

**Figure 1:**
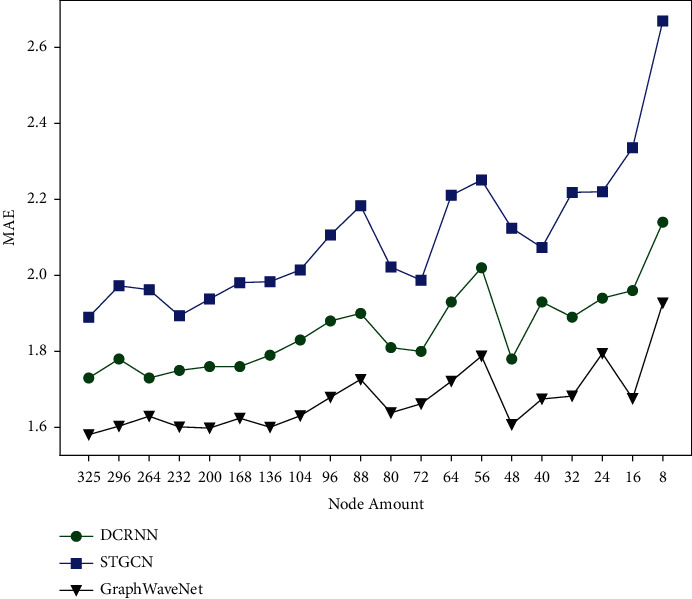
The performance of various existing methods on PeMS-BAY with different sparseness.

Existing approaches mainly focus on capturing the temporal and spatial dependencies based on dense spatial data. In the real world, there are not enough available detectors in some regions due to underdeveloped transportation or abnormal conditions (equipment maintenance, extreme weather, etc.) [16]. As the experiment shown in Figure 1, with the decrease of detector numbers, the prediction results of existing models turn to be worse. The reason is that most existing methods [5, 7, 17] only calculate the spatial dependence once in each module, and the attention mechanism is not fully utilized. Therefore, when the detectors are insufficient or the adjacent detectors are far away, the spatial correlation features cannot be fully captured by existing methods. In this article, we propose a novel deep learning architecture to address this problem. To the best of our knowledge, this work is the first to bring out the spatially sparse data problem.

To address the problem, we propose a novel framework called **M**ulticomponent **S**patial-**T**emporal **G**raph **A**ttention **C**onvolutional **N**etwork (MSTGACN), which consists of three relatively independent modules; each module is composed of multiple spatial-temporal graph attention convolution blocks to capture spatial correlations efficiently in the case of spatially sparse data. We sample a part of detector data from two public datasets, METR-LA and PeMS-BAY [7], and we construct a sparse dataset by sampling detectors in district-7 of Caltrans Performance Measurement System (PeMS). We evaluate MSTGACN on three sparse datasets, and experimental results demonstrate that MSTGACN outperforms existing methods.

Overall, the contributions of our work can be summarized as follows:

1. We propose a spatial-temporal graph attention convolution block consisting of dilated causal convolution, graph convolution layer, and graph attention layer. The parameters of two GAT layers are shared in one block. MSTGACN can more effectively capture the spatial-temporal features in the case of spatially sparse data.
2. We adopt two strategies to capture multiple periodic information effectively. First, day-of-week information and time-of-day information are extracted as additional features. Second, the input data and the model are divided into three components, which are used to capture the weekly, daily, and recent periodic features of data.
3. We bring out the problem of spatially sparse data in traffic prediction. Moreover, we evaluate our model MSTGACN on three real-world sparse datasets. Experimental results validate that the proposed model is superior to existing methods in the case of spatially sparse data.

To better present our work, the rest of this article is arranged as follows. We describe related works and the task definition in Section 2 and Section 3. Then, our method will be detailed and introduced in Section 4. We present our experimental results in Section 5. Finally, we conclude the article in Section 6.

## 2. Related Work

### 2.1. Temporal Modeling

Traffic prediction is a typical spatial-temporal sequence forecasting problem. Existing methods can roughly be categorized into two classes, namely, temporal modeling and spatial modeling. From the time series perspective, RNN and its variants have been proven to be able to extract temporal information. Recently, people have proposed many RNN-based models [18–22] in traffic prediction whose performance is superior to traditional statistical methods [23–25] and machine learning models [26, 27]. However, when the sequence length is long, the RNN-based model becomes inefficient and its gradients may explode. On the contrary, CNN has advantages of parallel calculation and stable gradient. Therefore, a CNN-based model [17] has been proposed to capture temporal dependencies. Besides, some methods [5, 28, 29] borrowed dilated causal convolution from the speech processing field to expand the receptive field. To capture periodic information of the time series, the authors of [8] constructed three different time series segments as the input to capture the periodic features in traffic data, but they did not utilize the time period information of each sample data, such as “time of day” and “day of week.”

### 2.2. Spatial Modeling

For spatial modeling, some previous methods [30–32] converted the road network at different times to a regular 2D grid and utilized traditional convolution to capture spatial correlations, while the non-Euclidean correlations in road networks have been ignored. Recent studies further explored the effectiveness of GCN in modeling non-Euclidean spatial structure; this is more in line with the structure of the road network in the real world. Many researchers proposed new approaches for effective spatial modeling based on GCN. Yu et al. [17] proposed Spatial-Temporal GCN, which was entirely composed of convolution structure in spatial and temporal dimensions. Li et al. [7] proposed Diffusion Convolutional Recurrent Neural Network and applied bidirectional random walks on graphs to capture the spatial dependency. Wu et al. [5] also adapted diffusion convolution, but they developed a novel adaptive dependency matrix to capture the hidden spatial dependency, which did not depend on prior knowledge. In these methods, the adjacency matrix represents the relationship between the nodes, but edges are much more complicated and interact with each other. Chen et al. [33] constructed the edgewise graph according to various edge interaction patterns and implemented the interactions between nodes and edges using bicomponent graph convolution. However, we found that the datasets used by the existing methods have a commonality. The nodes are relatively dense in space, and the adjacent nodes belong to the same road and are close to each other, so there is an obvious upstream and downstream relationship. For secondary roads in cities or roads in villages, the collection equipment is not as dense as the main roads in big cities. The abnormal equipment will also cause the problem of sparse node distribution. To study the traffic flow prediction problem in the sparse scenario, we sampled the existing datasets and constructed a new dataset with sparse points.

### 2.3. Attention Mechanism

The core idea of the attention mechanism is to dynamically focus on the most crucial information based on the input data. A large number of people have proposed attention-based models to solve traffic forecasting problems. Yin et al. [34] applied an internal attention mechanism to capture the interactions among multiple time series and a dynamic neighborhood-based attention mechanism to model the complex spatial correlations. Guo et al. [8, 35] applied temporal attention and spatial attention to capture dynamic spatial-temporal correlations. To stabilize the learning process, the researchers [36] replaced the traditional attention mechanism with a multi-head attention mechanism. Velickovic et al. [13] employed an attention mechanism into graph structure to dynamically adjust the importance of adjacent nodes. Guo et al. [28, 37] replaced GCN with graph attention networks (GAT) and [37] used meta knowledge to generate weights of GAT. GAT has achieved or matched state-of-the-art results across several benchmarks for graph-related tasks [38]. Considering that the spatial correlations are difficult to capture in the case of spatially sparse data, we employ multiple-stacked GAC blocks for better relation exploitation and prediction, which contains one GCN layer and one GAT layer in each block. The application of GAT with shared parameters in the block may also help alleviate the oversmoothing of GCN.

## 3. Preliminaries

The task of traffic prediction is to predict future traffic conditions (e.g., speed and volume) based on the historical traffic measurements of sensors in the road network. We define the road network as a weighted graph *G*=(*V*, *E*, *A*) with *N* nodes, where *V* is the set of nodes, *E* is the set of edges indicating the connectivity between the nodes, *A* ∈ *ℝ*^*N*×*N*^ is the weighted adjacency matrix of graph *G*. Suppose $\tilde{V}$ is the subset of *V*, indicating some nodes in the graph could not be used as input data when corresponding sensors are abnormal in the road network.

Problem. Given traffic data over past *P* time slices, the traffic data observed on *G* could be denoted as *X* ∈ *ℝ*^*N*′×*M*×*P*^(*N*′ ≤ *N*), where *M* is the number of traffic interests (e.g., traffic volume and traffic speed). When *N*′ ≪ *N* represents that the data are sparse, the goal of this task is to predict the traffic data (speed or flow) of the next *T*_*P*_ time steps, denoted as *Y*=(*Y*_1_, *Y*_2_,…, *Y*_*T*_*P*__) ∈ *ℝ*^*N*′×*T*_*P*_^.

## 4. Materials and Methods

In this section, we first present the overall framework of the MSTGACN and the method to capture periodic temporal information; then, we describe the spatial-temporal graph attention convolution (ST-GAC) block of our framework. Finally, we present the multicomponent fusion method.

### 4.1. The Architecture of MSTGACN

**Figure 2:**
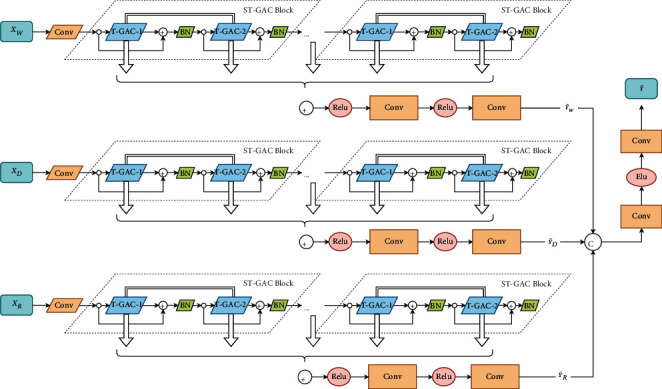
The architecture of our proposed MSTGACN.

As shown in Figure 2, MSTGACN proposed in this article consists of three independent components with the same structure, which are designed to model the recent, daily-periodic, and weekly-periodic dependencies of the data. Each component is composed of a convolution layer, several stacked ST-GAC blocks, and an output block. The headmost convolution layer captures the correlations between input features and generates multiple feature maps. The ST-GAC block models the spatial-temporal dependencies and each ST-GAC block is skip-connected to avoid oversmoothing. The outputs of the three components $\tilde{Y_{R}}$, $\tilde{Y_{D}}$,and $\tilde{Y_{W}}$ are fused into the final output $\tilde{Y}$ by the multicomponent fusion module.

### 4.2. Details about Three Time Series Segments

The spatial-temporal correlations vary with different periods. We adopt two strategies to capture multiple types of periodic information effectively. Firstly, we construct two metafeatures: “time of day” and “day of week” as external attributes. These additional features will be concatenated with original input data along the feature axis. Secondly, We intercept three time series segments ($\tilde{T_{R}}$, $\tilde{T_{D}}$, and $\tilde{T_{W}}$) along the temporal dimension to construct the input of recent, daily-period, and weekly-period components, respectively. Suppose the sampling frequency is *q* times per day. The current time is denoted as *t*_0_; *T*_*P*_ is the length of the sequence to be predicted. *T*_*R*_, *T*_*D*_, and *T*_*W*_ represent the length of input data for different components, and they are all integer multiples of *T*_*P*_. The details of the three segments are as follows.

$\tilde{T_{R}}=\left(X_{t_{0}-T_{R}+1},X_{t_{0}-T_{R}+2},\ldots ,X_{t_{0}}\right)\in {\mathbb{R}}^{N^{\prime }\times M\times T_{R}}$ . The adjacent sequence is closest to the period to be predicted. The traffic data (speed, flow) at a specific location change continuously with time. Thus, the data to be predicted will be affected by the data of the previous period.

$\tilde{T_{D}}=\left(X_{t_{0}-\left(T_{D}/T_{P}\right)*q+1},\ldots ,X_{t_{0}-\left(T_{D}/T_{P}\right)*q+T_{P}},\ldots ,X_{t_{0}-q+1},\ldots ,X_{t_{0}-q+T_{P}}\right)\in {\mathbb{R}}^{N^{\prime }\times M\times T_{D}}$ . It consists of the segments in the past few days at the same time period. This segment is used to provide information to model daily periodicity.

$\tilde{T_{W}}=\left(X_{t_{0}-7*\left(T_{W}/T_{P}\right)*q+1},\ldots ,X_{t_{0}-7*\left(T_{W}/T_{P}\right)*q+T_{P}},\ldots ,X_{t_{0}-7*q+1},\ldots ,X_{t_{0}-7*q+T_{P}}\right)\in {\mathbb{R}}^{N^{\prime }\times M\times T_{W}}$ . It is composed of the segments in the last few weeks, which have the same week attributes and the time intervals as the predicting period. This segment is constructed for modeling the weekly periodicity.

The three components share the same network structure described in the next section. The output of each component is denoted as $\tilde{Y_{R}}$, $\tilde{Y_{D}}$ and $\tilde{Y_{W}}$ respectively. These three outputs are merged by the multicomponent fusion module to obtain the final prediction result.

### 4.3. ST-GAC Block

**Figure 3:**
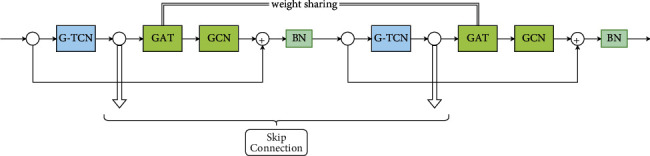
The architecture of ST-GAC block.

We first construct a GAC block, which contains a GAT layer and a GCN layer. The ST-GAC block is composed of two G-TCN blocks and two GAC blocks, as shown in Figure 3. Moreover, the two GAT layers in the same ST-GAC block share the same weights. The G-TCN block is used for capturing the temporal dependencies. The GAC layer is used to learn the correlations between nodes in the case of spatially sparse data. To make Figure 2 more concise, we combine a G-TCN layer and a GAC layer as a T-GAC module.

#### 4.3.1. Gated Temporal Convolution Layer

Inspired by Graph WaveNet [5], we adopt dilated causal convolution (TCN) to capture the temporal dependencies. Compared with the traditional 1D convolution, the dilated casual convolution skipped a fixed step to perform the convolution operation. Through stacking multiple dilated casual convolution layers, it is possible to make the receptive field increase exponentially. Meanwhile, when processing input sequences of the same length, the number of parameters in dilated convolution is less, and the training speed is faster than RNN. Because the spatial distribution of observation points is scattered and far apart, we believe that the relationship between observation points has a delay effect of one hour or even longer, and dilated convolution has better expansibility.

To learn the temporal information better, we utilize a gated mechanism based on the dilated casual convolution (G-TCN) to control information flow. Suppose *X* ∈ *ℝ*^*N*′×*M*×*P*^ is the input data; the output of a gated convolution is as follows:

(1) $$\begin{array}{c} h=\tanh\left(\Theta_{1}⋆X+b\right)⊙\sigma \left(\Theta_{2}⋆X+c\right), \end{array}$$

where Θ, *b*, and *c* are learning parameters, ⋆ is the convolution operator, ⊙ is the elementwise product, and *σ* is an activation function, which controls the ratio of information flow to the next layer.

#### 4.3.2. Graph Attention Convolution Block

Because GAT allows for aggregating information from other nodes by assigning different importance and GCN is an efficient variant of the convolutional neural network, which could be used in a non-Euclidean spatial structure, we construct a graph attention convolution block (GAC) with a GAT layer and a GCN layer to learn the spatial dependencies. In this work, we adopt the GCN layer proposed in Graph WaveNet to further model hidden spatial dependencies based on GAT, and the GCN formulation is as follows:

(2) $$\begin{array}{c} Z=\sum_{k=0}^{K}P_{f}^{k}{\text{XW}}_{k1}+P_{b}^{k}{\text{XW}}_{k2}+{\tilde{A}}_{apt}^{k}{\text{XW}}_{k3}, \end{array}$$

where ${\tilde{A}}_{apt}^{k}\in {\mathbb{R}}^{N^{\prime }\times N^{\prime }}$ represents the normalized adjacency matrix with self-loops, *X* ∈ *ℝ*^*N*′×*D*^ denotes the input data, *N*′ is the number of available nodes, *D* represents the characteristic dimension, *W* ∈ *ℝ*^*D*×*M*^ is the learning parameters, and *Z* ∈ *ℝ*^*N*′×*M*^ denotes the output. If the graph is directed, then the diffusion process has two directions. Let *P*_*f*_^*k*^ represent the forward direction, *P*_*b*_^*k*^ represent the backward direction, and *k* represent the order of diffusion. It is worth noting that when the road network is denoted as an undirected graph, equation (2) will be changed into the following:

(3) $$\begin{array}{c} Z=\sum_{k=0}^{K}P^{k}{\text{XW}}_{k1}+{\tilde{A}}_{apt}^{k}{\text{XW}}_{k2}. \end{array}$$

### 4.4. Multicomponent Fusion

In this section, we discuss how to integrate the outputs of the three components, $\tilde{Y_{R}}$, $\tilde{Y_{D}}$, and $\tilde{Y_{W}}$. In the multicomponent fusion block, $\tilde{Y_{R}}$, $\tilde{Y_{D}}$, and $\tilde{Y_{W}}$ are concatenated along the feature axis and regarded as feature vectors of different spatial-temporal dependencies. Then, we use two convolution layers with the ELU activation function to learn correlations of three components and the characteristics of each prediction time step. The outputs of the three components are fused as follows:

(4) $$\begin{array}{c} \tilde{Y}=W_{2}*ELU\left(W_{1}*\left[\tilde{Y_{R}}\left\|\tilde{Y_{D}}\right\|\tilde{Y_{W}}\right]\right), \end{array}$$

where ‖ means concatenation operation and *∗* is the convolution operation.

## 5. Experiments and Results

### 5.1. Datasets

We verify MSTGACN on three traffic datasets, METR-LA, PeMS-BAY, and PeMSD7-sparse. To test the performance of various models in the case of sparse data points, we reconstruct datasets with different degrees of sparsity by selecting sensors. For METR-LA, we select 24, 32, 40, 48, 56, 64, 72, 80, 88, 96, 104, 136, 168, and 200 nodes to reconstruct 14 datasets with different spatial densities. For PeMS-BAY, besides the amounts of sensors used in METR-LA, we also selected 232, 264, and 296 nodes to reconstruct 17 datasets. We adopt the same data preprocessing procedures as in [7]. In both datasets, a time step denotes 5 minutes and the data are normalized via the *Z*-Score method.

**Figure 4:**
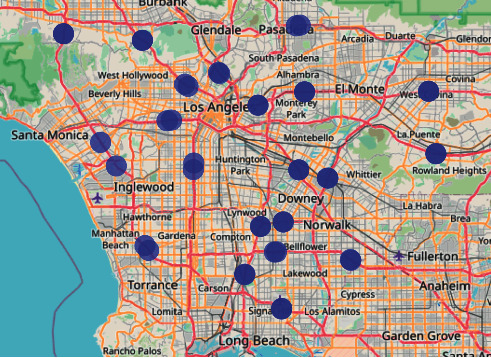
The distribution of selected sensors in PeMSD7-sparse.

 
**METR-LA**: This dataset records four months of traffic speed data ranging from Mar 1st, 2012, to June 30th, 2012, including 207 sensors on the highways of Los Angeles County.
 
**PeMS-BAY**: This dataset is collected from the California Transportation Agencies (CalTrans) Performance Measurement System (PeMS). It records six months of statistics on traffic speed ranging from Jan 1st, 2017, to May 31st, 2017, including 325 sensors in the Bay Area [39].
 
**PeMSD7-sparse**: We selected 42 sensors in Los Angeles and collected two months of data ranging from Mar 1st, 2012, to July 2nd. The selected 42 sensors can be divided into 21 pairs; the two sensors in a pair are very close but face opposite directions. It can be considered that there are 21 nodes in the undirected graph, and each node contains traffic data in two directions. We use the Euclidean distance to calculate the distance between two nodes. The sensor distributions are visualized in Figure 4.

In all of these datasets, we aggregate traffic data into 5 minutes and apply Z-Score normalization. The datasets are split in chronological order with 70% for training, 10% for validation, and 20% for testing.

### 5.2. Baselines

We compare MSTGACN with the following models.

 
**HA**: Historical Average that models the traffic flow as a seasonal process and uses a weighted average of previous seasons as the prediction.
 
**VAR** [40]: Vector Auto-Regression, a more advanced time series model that captures the pairwise relationships among all traffic flow series.
 
**DCRNN** [7]: Diffusion Convolutional Recurrent Neural Network that integrates diffusion convolution with recurrent neural networks.
 
**Graph WaveNet** [5]: a convolution network architecture that combines graph convolution with dilated casual convolution and introduces a self-adaptive adjacency matrix.
 
**STGCN** [17]: A spatial-temporal graph convolution model to predict traffic speed.
 
**ASTGCN** [8]: Attention-Based Spatial-Temporal Graph Convolutional Networks, which combines the spatial-temporal attention mechanism and the spatial-temporal convolution.
 
**ST-MetaNet** [37]: a model with graph attention networks (GAT), using metaknowledge to generate weights of GAT.

### 5.3. Experimental Settings

Our experiments are conducted on a 64-bit Linux Server with one Intel(R) Core(TM) i7-7800X CPU @ 3.50 GHz and one NVIDIA Titan Xp GPU card. All the tests use 60 minutes as the historical time window. In other words, 12 data points are used to predict the traffic data in the next 5, 15, and 30 minutes. To cover the input sequence length, we use four ST-GAC blocks, and dilated factors of the two T-GAC blocks are set as 1 and 2, respectively. We adopt Adam optimizer to train our model. The initial learning rate is set as 0.001. The dropout rate is 0.5 and 0.3 in GCN and GAT; we set the output dimensions of the GAT layer and GCN layer to be 32, respectively. We use equation (2) as our graph convolution layer and the diffusion steps *K* is set as 2. The adjacency matrix is constructed by road network distance with a thresholded Gaussian kernel.

(5) $$\begin{array}{c} A_{v_{i},v_{j}}=\left\{\begin{array}{cc} \exp\left(-\frac{d_{v_{i},v_{j}}^{2}}{\sigma^{2}}\right), & \text{if }\exp\left(-\frac{d_{v_{i},v_{j}}^{2}}{\sigma^{2}}\right)\geq \varepsilon ,\\ 0, & \text{otherwise,} \end{array}\right. \end{array}$$

where *d*_*v*_*i*_,*v*_*j*__ is the distance between point *i* and point *j*, *σ* is the standard deviation, and*ϵ* is a threshold value to control the matrix sparse degree, and we set *ε*=0.1 in our model.

To evaluate the performance of different methods, we evaluate MSTGACN, HA, VAR, DCRNN, STGCN, ST-MetaNet. and Graph WaveNet. For these seven models on METR-LA, PeMS-BAY, and PeMSD7-sparse, we adopt Mean Absolute Errors (MAE) and Root Mean Squared Errors (RMSE) as the evaluation metrics.

## 6. Quantitative Experimental Results

Tables 1 and 2 demonstrate the average results of MSTGACN and the baseline methods on PeMS-BAY and METR-LA with a different number of nodes. It can be seen that although MSTGACN is second only to Graph WaveNet on the complete dataset, as the number of nodes decreases, the performance of our model gradually exceeds other methods. Tables 1 and 2 show that the performances of STGCN are the worst among models based on deep learning. It may be because STGCN defines the road network as an undirected graph, and these two datasets are defined as directed graphs in DCRNN [7]. The lack of direction leads to a decrease in STGCN performance. To verify this view and further test the validity of MSTGACN, we evaluate these models on the PeMSD7-sparse dataset.

Table 3 gives the results of MSTGACN and the baseline methods for 5 minutes, 15 minutes, and 30 minutes ahead prediction on the PeMSD7-sparse dataset. We observe the following:

1. On the traffic dataset with scattered location distribution, whether short-term prediction within 5 minutes or a mid-term prediction of 30 minutes, the prediction effects of HA and VAR are bad. It is because HA does not mine the spatial or temporal features of data. Because of VAR's limited modeling ability and the inability to learn mid- and long-term changes, it does not perform well in mid- and long-term prediction.
2. The accuracy of STGCN and ASTGCN is lower than that of Graph WaveNet and the proposed method. By comparing their original datasets, it can be found that there are apparent up-down relationships in the datasets used in the two articles. Therefore, in the case of scattered point distribution, STGCN and ASTGCN cannot effectively learn the spatial-temporal dependencies in the data. The method of capturing the spatial-temporal dependencies based on the attention mechanism proposed in ASTGCN is conducive to long-term prediction. Therefore, under the prediction time of 30 minutes, the performance of STGCN is better than ASTGCN.
3. Single Recent is a degraded version of MSTGACN, which only has one recent component. Due to the designed GAC block, even the single-component model, its performance is better than baselines.

The proposed model uses the GAC block to learn the spatial dependencies, and the multicomponent structure helps capture the correlations under different periods. Thus, our MSTGACN achieves the best performance in PeMSD7-sparse in terms of all evaluation metrics. To verify the effectiveness of the multicomponent division, we investigate our model with different component settings. As shown in Table 3, we find that the performance of the degraded model with only the weekly component or daily component is not good. It can be considered that it is difficult to learn the temporal and spatial features of spatial-temporal data using only these two periods of data, which indicates that spatial-temporal features are dynamically changing, so it is hard to make accurate predictions for future data using historical data that are far apart. The single recent model performs much better than the single weekly and single daily model, and the triple recent model gets better performance than the single recent model. It could be considered that the components in the triple recent model are combined according to the bagging method, which can effectively improve the performance of ensemble models. The proposed model, which consists of recent, daily, and weekly components, achieves the lowest predicting errors.

### 6.1. Comparative Experiment of GAC Block

In traffic prediction tasks, different spatial nodes are correlated. Accurately capturing the correlations between sensors in road networks is necessary to predict the traffic data. Because the PeMS dataset is a highway dataset, there are many adjacent points on the same road when the points are densely distributed. These points have an obvious upstream and downstream relationship. When the number of nodes decreases, there are few adjacent points located on the same road, and there are many intersections between different nodes. Therefore, the upstream and downstream relationship between the points is not obvious and the spatial correlation between different points decreases. Because there are multiple intersections between different detectors, we think that the single use of GAT or GCN is insufficient to capture the spatial relationship.

Before each GCN module, we used an extra GAT module. Although the original intention was to increase the model's ability by extracting features through GAT and summarizing information through GCN on sparse data, the application of this attention mechanism with shared parameters in the block could also help alleviate the oversmoothing of GCN. We did comparative experiments of different modules on PeMSD7-sparse. As shown in Table 4, GAT + GCN shows the best result.

## 7. Qualitative Experimental Results

### 7.1. Visual Comparison of MAE

**Figure 5:**
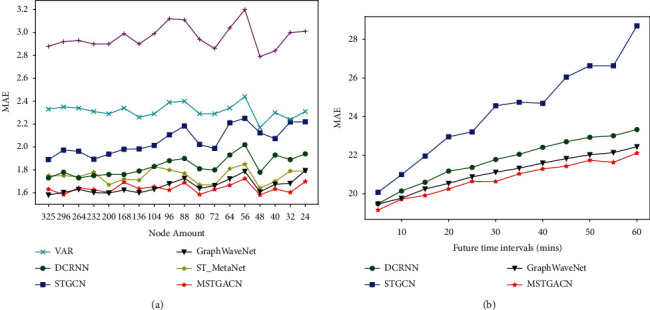
The performance of various existing methods. (a) Performance on sparse PeMS-BAY with different node amounts. (b) Performance of different methods in different forecasting duration.

Figure 5 demonstrates the average results on PeMS-BAY. It can be seen that although MSTGACN is second only to Graph WaveNet on the complete dataset, as the number of nodes decreases, the performance of our model gradually exceeds other methods. In order to study the influence of prediction time on model performance, the prediction time is gradually increased from 5 minutes to 1 hour at an interval of 5 minutes. As shown in the figure, the model proposed in this article has achieved good results in both short-term prediction and long-term prediction.

### 7.2. Visualization of Attention Matrix

**Figure 6:**
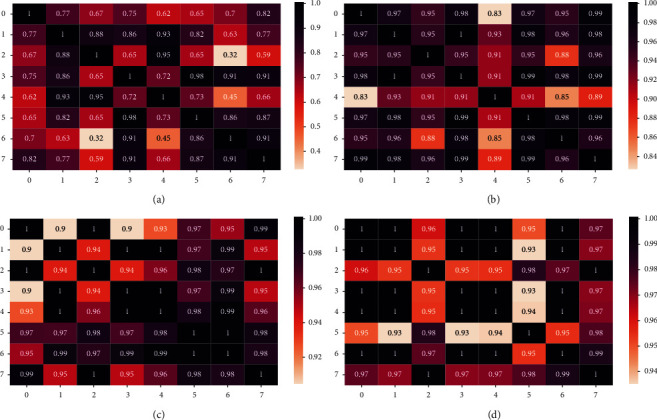
Input an hour data, the spatial attention matrix coefficients calculated by different GAT layers. (a) The first GAT calculation. (b) The second GAT calculation. (c) The penultimate GAT calculation. (d) The last GAT calculation.

To test the performance of stacked GAC blocks, we show different spatial attention matrices among detectors in the PeMS-BAY with 8 nodes. As shown in Figure 6, as the number of GAT computations increases, the spatial attention matrix coefficients also increase. This is reasonable since the stacked GAC blocks increase the receptive field and the distant points also could be highly correlated.

### 7.3. Visualization of Traffic Flow

**Figure 7:**
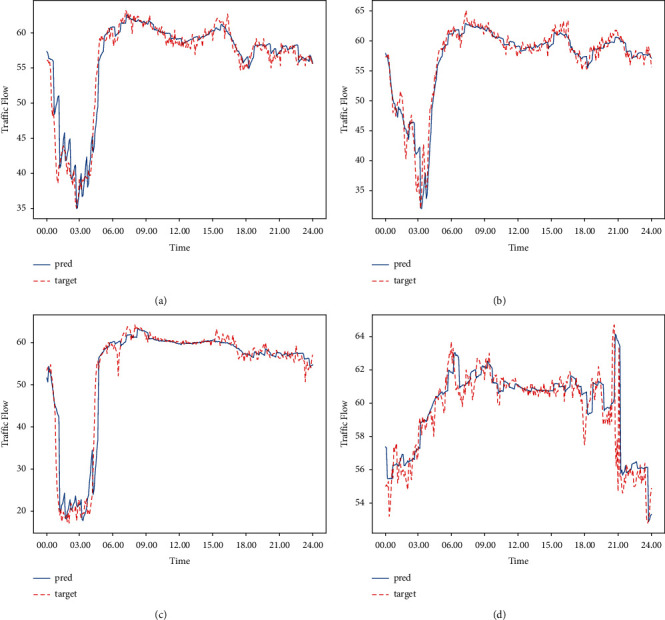
Comparison of the predicted traffic flow with the real data.

As shown in Figure 7, we selected four days of data for visual comparison and found that our model could predict the same data trends as the real data.

## 8. Conclusions

In this article, we propose a deep learning framework for traffic prediction in the case of spatially sparse data. The model combines dilated causal convolution, graph convolution layer, and the weight-shared graph attention layer. The parameters of two GAT layers are shared in one block to capture the spatial-temporal dependencies of traffic data in the case of sparse points. To capture the multiple periodic information, we extract the day-of-week and time-of-day information as additional features. Moreover, we divide the input data and model structure into three components. Experiments on three real-world datasets show that the predicting accuracy of our model is superior to baselines. In general, traffic data are affected by many external factors, like weather, events, and holidays. In the future, these external factors should be taken into consideration to improve the predicting performance in the case of spatially sparse data.

## Figures and Tables

**Table 1:** Performance comparison of different methods on PeMS-BAY with different number of nodes.

Model	24 nodes		56 nodes		88 nodes		232 nodes		325 nodes	
	MAE	RMSE	MAE	RMSE	MAE	RMSE	MAE	RMSE	MAE	RMSE
HA	3.01	6.15	3.20	6.37	3.11	6.08	2.90	5.67	2.88	5.99
VAR	2.31	4.52	2.44	4.55	2.40	4.44	2.31	4.14	2.33	4.12
DCRNN	1.94	4.46	2.02	4.69	1.90	4.25	1.75	3.87	1.73	3.89
STGCN	2.22	4.84	2.25	4.82	2.18	4.66	1.89	4.15	1.89	4.31
ST-MetaNet	1.79	4.35	1.85	4.32	1.77	4.13	1.78	4.16	1.75	4.09
GraphWaveNet	1.79	4.21	1.79	4.10	1.73	3.87	**1.60**	3.58	**1.58**	**3.54**
MSTGACN (ours)	**1.70**	**3.90**	**1.72**	**3.84**	**1.69**	**3.77**	1.63	**3.57**	1.63	3.58

**Table 2:** Performance comparison of different methods on METR-LA with different number of nodes.

Model	24 nodes		56 nodes		80 nodes		136 nodes		207 nodes	
	MAE	RMSE	MAE	RMSE	MAE	RMSE	MAE	RMSE	MAE	RMSE
HA	6.82	11.26	6.47	10.91	6.91	11.34	7.37	11.83	7.50	11.93
VAR	4.58	8.51	4.61	8.44	4.73	8.40	4.74	8.34	4.68	8.37
DCRNN	3.64	7.46	3.32	6.83	4.20	9.99	3.10	6.29	3.17	6.47
STGCN	6.06	9.35	6.03	9.11	6.03	9.12	6.09	9.08	3.65	7.46
ST-MetaNet	3.25	6.82	3.03	6.33	3.10	6.37	**3.00**	6.20	3.06	6.23
GraphWaveNet	**3.22**	6.52	**3.01**	6.14	**3.09**	**6.14**	3.02	**6.07**	**3.04**	**6.09**
MSTGACN (ours)	**3.22**	**6.47**	**3.01**	**6.09**	3.11	6.18	3.06	6.10	3.14	6.16

**Table 3:** Performance comparison of different methods on PeMSD7-sparse. Single Weekly, Single Daily, Single Recent, and Triple Recent are the degraded versions of MSTGACN.

Model	5 min		15 min		30 min	
	MAE	RMSE	MAE	RMSE	MAE	RMSE
HA	26.25	40.60	26.25	40.60	26.25	40.60
VAR	20.44	30.40	25.56	37.35	30.60	43.86
STGCN	20.14	29.72	23.43	34.54	26.70	39.03
ST-MetaNet	22.27	32.07	24.45	35.58	26.44	38.91
ASTGCN	21.00	30.55	23.61	34.67	25.98	37.96
DCRNN	19.69	29.40	21.83	33.20	23.48	35.87
Graph WaveNet	19.38	28.83	22.39	32.95	23.43	35.61
Single Weekly (ours)	26.79	40.84	26.46	40.68	26.40	40.50
Single Daily (ours)	26.84	40.68	26.59	40.43	26.68	40.46
Single Recent (ours)	19.23	28.74	21.28	32.29	22.85	34.67
Triple Recent (ours)	19.19	28.57	21.12	32.02	22.64	34.45
MSTGACN (ours)	**18.88**	**28.35**	**20.65**	**31.42**	**21.70**	**33.33**

**Table 4:** Performance comparison of different methods on PeMSD7-sparse. Single Weekly, Single Daily, Single Recent, and Triple Recent are the degraded versions of MSTGACN.

Module	5 min		15 min		30 min	
	MAE	RMSE	MAE	RMSE	MAE	RMSE
GAT	19.86	29.33	21.99	33.01	23.84	35.87
GCN	19.38	28.83	22.39	32.95	23.43	35.61
GCN + GAT	19.43	28.96	21.47	32.64	23.06	35.25
GAT + GCN	**19.23**	**28.74**	**21.28**	**32.29**	**22.85**	**34.67**

## Data Availability

Previously reported METR-LA and PeMS-BAY data were used to support this study and are available at https://dblp.org/rec/conf/iclr/LiYS018. These prior studies (and datasets) are cited at relevant places within the text as references. Previously reported PeMS-D7 data were used to support this study and are available at https://dblp.org/rec/conf/aaai/SongLGW20. These prior studies (and datasets) are cited at relevant places within the text as references.
